# CT appearances and classification of hepatic epithelioid hemangioendothelioma

**DOI:** 10.1186/s13244-023-01410-z

**Published:** 2023-04-01

**Authors:** Haidong Tan, Ruiquan Zhou, Hongwei Yu, Feng Teng, Shuang Si, Liguo Liu, Shiwei Yang, Dongdong Han, Xiaolei Liu

**Affiliations:** 1 Second Department of Hepatopancreatobiliary Surgery, China-Japan Friendship Hospital, 2 Yinghua Dongjie, Hepingli, Beijing, 100029 China; 2 Department of Radiology, China-Japan Friendship Hospital, Beijing, China; 3 Department of Radiation Oncology, China-Japan Friendship Hospital, Beijing, China

**Keywords:** Hepatic epithelioid hemangioendothelioma, CT, Hepatic tumors, Rare liver tumors

## Abstract

**Background:**

Hepatic epithelioid hemangioendothelioma (HEH) is extremely rare, and CT features have never been analyzed in a large group of patients.

**Methods:**

A retrospective study was designed to review the contrast-enhanced CT images of HEH patients. Intrahepatic lesions were categorized into three types: nodular, locally coalescent (coalescent lesion contained in one segment) or diffusely coalescent (coalescent lesion occupied more than one segment). CT features were compared among lesions of different sizes and patients with different lesion types.

**Results:**

A total of 93 HEH patients were included in this study, and 740 lesions were analyzed. The results of per-lesion analysis showed that medium lesions (2–5 cm) had the highest rate of lollipop sign (16.8%) and target-like enhancement (43.1%), while lesions in large group (> 5 cm) had the highest rate of capsular retraction (38.8%) and vascular invasion (38.8%). The differences on enhancement pattern and the rates of lollipop sign and capsular retraction were significant among lesions of different sizes (*p* < 0.001, respectively). The results of per-patient analysis showed that patients in locally coalescent group had the highest rates of lollipop sign (74.3%) and target sign (94.3%). All patients in diffusely coalescent group had capsular retraction and vascular invasion. CT appearances of capsular retraction, lollipop sign, target sign and vascular invasion differed significantly among patients with different lesion types (*p* < 0.001, *p* = 0.005, *p* = 0.006 and *p* < 0.001, respectively).

**Conclusion:**

CT features variated among HEH patients with different lesion types, and radiological appearances of HEH should be classified into nodular type, locally coalescent type and diffusely coalescent type.

## Key points

1. CT appearances of HEH differed significantly among lesions of different sizes.
2. HEH lesions were categorized into three types: nodular, locally and diffusely coalescent.
3. CT characteristics of HEH variated greatly among the three lesion types.

## Introduction

Epithelioid hemangioendothelioma (EH) is an extremely rare tumor, which originates from the vascular endothelium, and liver is the most commonly primary site [1]. The biological behavior of hepatic epithelioid hemangioendothelioma (HEH) is deemed between angiosarcoma and hemangioma, but the malignancy variates greatly among patients [2]. The tumor is indolent for most patients and could regress spontaneously without any treatment in some cases [3]. Usually, multiple intrahepatic lesions are occasionally detected by ultrasonography or computed tomography (CT) examination with no symptom [4]. Moreover, extrahepatic lesions are always simultaneously found in many HEH patients [4, 5]. Singular intrahepatic lesion without metastasis is a rare scenario in HEH. Currently, due to the rarity of the disease, no standard treatment paradigm has been established yet. Surgical resection and liver transplantation have been retrospectively studied to be effective for HEH [6, 7]. However, radical surgery or liver transplantation was impossible for most HEH patients accompanied with multiple intrahepatic and extrahepatic metastases at the time of diagnosis [4, 5, 8]. Interferon-alpha 2b (IFN-a 2b) as an immunotherapy has also been used for the treatment of EH, and our previous research showed IFN-a 2b had good efficacy for HEH [5, 9].

Due to the rarity, HEH is often misdiagnosed as cholangiocarcinoma or metastatic tumor by magnetic resonance imaging (MRI) or CT. Thus, analyzing and summarizing the radiological features of HEH are very important. Several studies have reported that subcapsular lesions, coalescent lesions, capsular retraction, target sign and lollipop sign are the radiological features of HEH [10–13]. However, considering the limited cases, the results of these studies failed to provide a comprehensive description of HEH on MRI or CT scans, based on a large group of patients. In 2021, we reported our study on MRI appearances of 57 HEH patients, which was the largest cohort at that time, and the results showed that MRI appearances differed significantly among lesions of different sizes [8]. Until September 2022, a total of 135 HEH patients are regularly followed up by our team, which is the largest single institution cohort. Thus, this study aimed to investigate the CT features of HEH on the largest group ever reported and explore the differences of radiological characteristics among patients with different lesion types.

## Patients and methods

### Patients

A retrospective study was conducted in a group of HEH patients who were histologically diagnosed from March 2014 to September 2022. All their radiological data before and after each treatment were retrospectively collected. This study aimed to analyze the CT appearances of HEH patients by retrospectively reviewing the CT images at the time of diagnosis. The inclusion criteria were as follows: (1) patients who underwent liver biopsy or surgical liver resection for pathological diagnosis; (2) patients who underwent contrast-enhanced CT scan within 30 days prior to surgery or biopsy. The exclusion criteria were as follows: (1) patients with incomplete CT images or CT images of insufficient quality; (2) patients who had received any kind of treatment prior to the CT scan; and (3) coexistence with other liver malignancies or severe hepatic steatosis. All the included HEH patients signed the consent forms to authorize the research of using their radiological images and clinical data. This study was censored and approved by the Ethics Committee of China-Japan Friendship Hospital (Number: 2022-KY-099).

### CT imaging and analysis

Both unenhanced and contrast-enhanced CT images should be available for all the included patients. CT examinations should be performed on a spiral CT scanner, and data sets were reconstructed with slice thicknesses of 3–5 mm. Contrast-enhanced CT scans were obtained in all included patients following intravenous administration of contrast material with iodine content, including arterial phase, portal phase and equilibrium phase. All CT images were independently reviewed by two radiologists (HW.Y. with 15 years of experience and F.T. with 16 years of experience) with a specialty in abdominal imaging using a picture archiving and communication system (PACS) under the setting of abdominal window (window width 240 HU, window level 40 HU). The analysis of CT images was performed on both per-lesion and per-patient basis by both reviewers. While for patients with more than 10 intrahepatic lesions, only the ten largest lesions were evaluated. After independent image analysis, a joint review was held to reach a consensus when discordant opinions occurred between the two reviewers.

CT appearances of per-lesion were analyzed with following parameters: (1) lesion size (the maximum diameter); (2) lesion type (nodular, locally coalescent (coalescent lesion contained in one segment) or diffusely coalescent (coalescent lesion occupied more than one segment)); (3) contour (round, round-like, irregular or stripe-like); (4) lesion density compared with liver on unenhanced scan (low, equal or high); (5) subcapsular lesion (any portion of the lesion in contact with the liver capsule); (6) capsular retraction (adjacent liver surface was retracted toward the lesion); (7) lollipop sign (hepatic/portal vein and/or their tributaries/branches tapering and terminating at the edge of a well-defined peripherally lesion); (8) calcification (lesions showing point or nodular high density which was similar to the bone); (9) vascular invasion (hepatic/portal vein and/or their tributaries/branches penetrated lesions or was surrounded by lesions with or without intact vessel structure); (10) target sign on unenhanced scan (two or multiple concentric layered “target-like” appearance on unenhanced scan); (11) the pattern of enhancement on arterial, portal and equilibrium phases (none, ring-like, target-like, core or heterogenous). Locally or diffusely coalescent lesion which was coalesced by several lesions was analyzed as one lesion.

CT appearances of per-patient were analyzed with the following parameters: (1) number of lesions (singular, 2–10 or more than 10); (2) the type of largest lesion (nodular, locally coalescent or diffusely coalescent); (3) subcapsular lesion (at least one subcapsular lesion); (4) capsular retraction (at least one lesion with capsular retraction); (5) lollipop sign (at least one nodular lesion with lollipop sign on portal phase); (6) target sign (two or multiple concentric layered “target-like” appearance on any scan); (7) calcification (at least one lesion with calcification); (9) vascular invasion (at least one lesion with vascular invasion).

The clinical data of all included HEH patients were retrospectively collected, including symptom and liver function within 30 days prior to surgery or biopsy, and the existence of perihepatic effusion was also reviewed on CT scan.

### Statistical analysis

Categorical variables and qualitative CT features are described as frequencies and percentages. The Chi-square test (Pearson and continuity correction) and Fisher’s exact test were employed to evaluate differences between the groups. The measurement data with normal distributions were represented as means ± SDs and between-group comparisons were performed using the one-way ANOVA. The measurement data with abnormal distributions are expressed as medians ± inter-quartile range, and between-group comparisons were performed using non-parametric tests. Differences with p < 0.05 were considered significant. The data were analyzed using SPSS 24.0 (Chicago, IL, USA).

## Results

### General Information

**Fig. 1:**
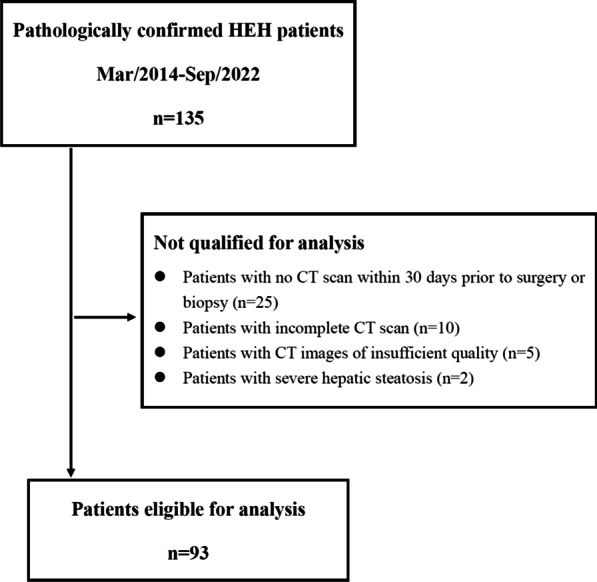
Patient flow chart for inclusion. A total of 135 pathologically confirmed hepatic epithelioid hemangioendothelioma (HEH) patients were regularly followed up in our center. Excluded were 25 patients with no CT scan within 30 days prior to surgery or biopsy, 10 patients with incomplete CT scan, 5 patients with CT images of insufficient quality and 2 patients with severe hepatic steatosis

From March 2014 to September 2022, a total of 135 pathologically diagnosed HEH patients were regularly followed up, and their detailed clinical and radiological data were retrospectively collected. All 57 patients in our previous report were also included in the current group of 135 HEH patients [8]. Forty-two patients not qualified for analysis were excluded. Ninety-three HEH patients were finally included in this study, including 23 patients reported in our previous study [8] (Fig. 1, Table 1). At the time of diagnosis, extrahepatic metastases were simultaneously detected in 48 (51.6%) patients, including 36 (38.8%) patients with metastases in one organ and 12 (12.9%) patients with metastases in multiple organs (Table 1). Ninety (96.8%) patients had multiple intrahepatic lesions, and only 3 (3.2%) patients had single intrahepatic lesion (Table 1).

**Table 1:** Demographic summary of included patients

Parameters	All patients (*n* = 93)
Age (years)	39.4 ± 13.9
Gender	
Male	42 (45.2%)
Female	51 (54.8%)
Diagnosed procedure	
Surgery	16 (17.2%)
Liver biopsy	77 (82.8%)
Number of intrahepatic lesions	
Single	3 (3.2%)
2–10	38 (40.9%)
More than 10	52 (55.9%)
Extrahepatic metastases	
Lung	32 (34.4%)
Bone	2 (2.2%)
Spleen	1 (1.1%)
Peritoneum	1 (1.1%)
Lung + bone	5 (5.4%)
Lung + peritoneum	2 (2.2%)
Lung + spleen	2 (2.2%)
Spleen + bone	2 (2.2%)
Brain + bone	1 (1.1%)

### CT appearances of HEH on per-lesion analysis

**Fig. 2:**
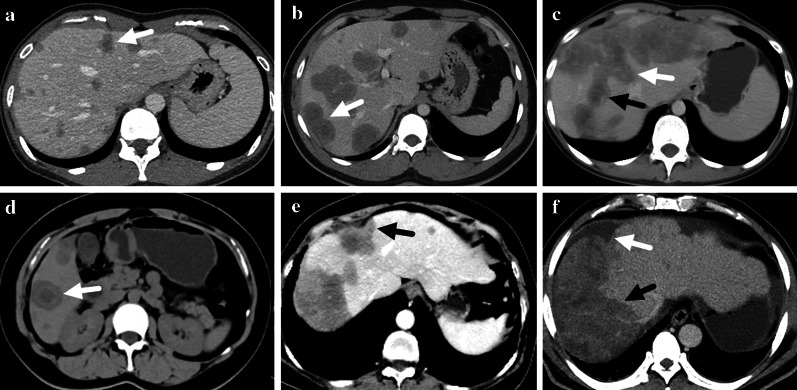
**a** A nodular lesion with target sign was marked with a white arrow. **b** A locally coalescent lesion with target sign was marked with a white arrow. **c** A diffusely coalescent lesion occupied multiple liver segments (marked with a white arrow). Target sign could also be found in the lesion (marked with a black arrow). **d** Target sign could be found on unenhanced CT scan (marked with a white arrow). **f** Capsular retraction caused by a subcapsular lesion could be detected (marked with a black arrow). **e** A diffusely coalescent lesion (marked with a black arrow) was accompanied with capsular retraction and perihepatic effusion (marked with a white arrow)

**Fig. 3:**
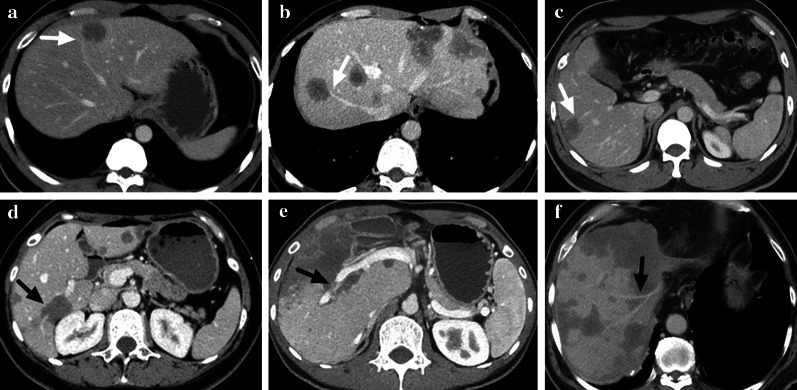
**a–c** Lollipop sign on portal phase of three HEH patients, which showed vessels terminated at the edge of a well-defined lesion (marked with white arrows). **d–f** Vascular invasion on portal phase of three HEH patients, which showed vessels penetrated lesions or was surrounded by lesions with or without intact vessel structure (marked with black arrows)

**Fig. 4:**
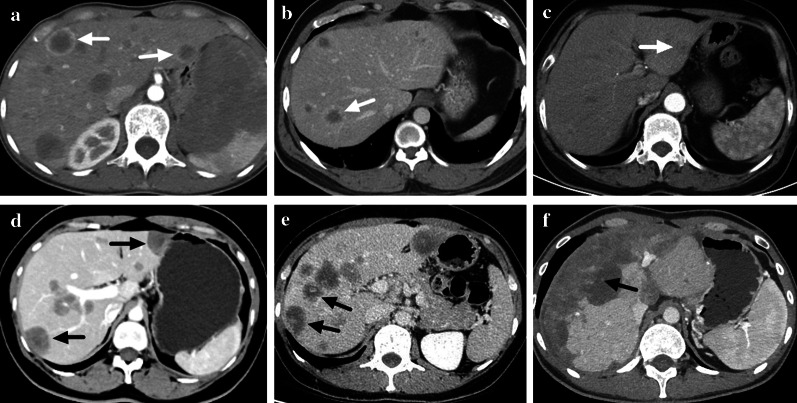
Enhancement patterns of HEH lesions on contrast-enhanced CT scan. **a** Ring-like enhancement on arterial phase (marked with white arrows). **b** Ring-like enhancement on portal phase (marked with a white arrow). **c** Target-like enhancement on arterial phase (marked with a white arrow). **d** Target-like enhancement on portal phase (marked with black arrows). **e** Core enhancement on portal phase (marked with black arrows). **f** Heterogenous enhancement on portal phase (marked with a black arrow)

For the 41 (44.1%) patients with 1–10 lesions, all their intrahepatic lesions were analyzed, while for the 52 (55.9%) patients with more than 10 lesions, only the 10 largest lesions were analyzed. Finally, a total of 740 intrahepatic lesions were reviewed and analyzed. According to the size, the analyzed lesions were categorized into three groups: small (< 2 cm, *n* = 221, 29.9%), medium (2–5 cm, *n* = 452, 61.1%) and large (> 5 cm, *n* = 67, 9.1%). Most of the analyzed lesions were nodular (80.7%), and the rate of locally and diffusely coalescent lesions increased as the size increased with significant differences among groups (*p* < 0.001) (Fig. 2, Table 2). The contour of most lesions was round (37.7%) or round-like (40.3%), while the rate of irregular and stripe-like lesion also increased as the size increased with significant differences among groups (*p* < 0.001) (Table 2). The density on plain scan was low for 96.1% lesions and 10.8% lesions showed target sign on plain scan (Fig. 2). The rate of subcapsular lesion and capsular retraction differed significantly among groups (*p* < 0.001) and increased as the size increased (Fig. 2, Table 2). Lollipop sign was detected in 12.6% lesions, and medium lesions had the highest rate compared with small and large groups (*p* < 0.001) (Fig. 3, Table 2). The rate of vascular invasion was highest in large group (38.8%) with significant difference compared with small and medium groups (*p* < 0.001) (Fig. 3, Table 2). For lesions in small group, the most common pattern of enhancement was none enhancement (arterial: 85.5%; portal: 74.2%; equilibrium: 73.3%). The rate of target-like enhancement was the highest in medium group (arterial: 35.4%; portal: 43.1%; equilibrium: 42.3%) (Fig. 4). The lesions in large group had the highest rate of heterogenous enhancement (arterial: 53.7%; portal: 61.2%; equilibrium: 61.2%) (Fig. 4). The enhancement pattern had significant difference among groups (*p* < 0.001) (Table 2). The rate of calcification was only 2.0% in all the lesions and large group had the highest rate (9.0%).

**Table 2:** Comparison of CT appearances of HEH lesions among different size groups

Parameters	Total (*n* = 740)	Small (*n* = 221)	Medium (*n* = 452)	Large (*n* = 67)	*p* value
Size (cm)	3.1 ± 2.4	1.4 ± 0.4	3.2 ± 1.2	8.4 ± 4.0	< 0.001
Lesion Type					
Nodular	597 (80.7%)	220 (99.5%)	370 (81.9%)	7 (10.4%)	< 0.001
Locally Coalescent	122 (16.5%)	1 (0.5%)	81 (17.9%)	40 (59.7%)	
Diffusely Coalescent	21 (2.8%)	0	1 (0.2%)	20 (29.9%)	
Contour					
Round	279 (37.7%)	139 (62.9%)	136 (30.1%)	4 (6.0%)	< 0.001
Round-like	298 (40.3%)	72 (32.6%)	217 (48.0%)	9 (13.4%)	
Irregular	127 (17.2%)	8 (3.6%)	76 (16.8%)	43 (64.2%)	
Strip-like/Flaky	36 (4.9%)	2 (0.9%)	23 (5.1%)	11 (16.4%)	
Density on Plain Scan					
Low	711 (96.1%)	195 (88.2%)	449 (99.3%)	67 (100%)	< 0.001
Equal	29 (3.9%)	26 (11.8%)	3 (0.7%)	0	
Target Sign on Plain Scan					
Yes	80 (10.8%)	0	68 (15.0%)	12 (17.9%)	< 0.001
No	660 (89.2)	221 (100%)	384 (85.0%)	55 (82.1%)	
Subcapsular Lesion					
Yes	239 (32.3%)	31 (14.0%)	164 (36.3%)	44 (65.7%)	< 0.001
No	501 (67.7%)	190 (86.0%)	288 (63.7%)	23 (34.3%)	
Capsular Retraction					
Yes	88 (11.9%)	3 (1.4%)	59 (13.1%)	26 (38.8%)	< 0.001
No	652 (88.1%)	218 (98.6%)	393 (86.9%)	41 (61.2%)	
Lollipop Sign					
Yes	93 (12.6%)	11 (5.0%)	76 (16.8%)	6 (9.0%)	< 0.001
No	647 (87.4%)	210 (95.0%)	376 (83.2%)	61 (91.0%)	
Vascular Invasion					
Yes	51 (6.9%)	0	25 (5.5%)	26 (38.8%)	< 0.001
No	689 (93.1%)	221 (100%)	427 (94.5%)	41 (61.2%)	
Calcification					
Yes	15 (2.0%)	4 (1.8%)	5 (1.1%)	6 (9.0%)	< 0.001
No	725 (98.0%)	217 (98.2%)	447 (98.9%)	61 (91.0%)	
Enhancement Pattern					
Arterial					
None	398 (53.8%)	189 (85.5%)	196 (43.4%)	13 (19.4%)	< 0.001
Ring	56 (7.6%)	23 (10.4%)	33 (7.3%)	0	
Target	186 (25.1%)	8 (3.6%)	160 (35.4%)	18 (26.9%)	
Core	13 (1.8%)	1 (0.5%)	12 (2.7%)	0	
Heterogeneous	87 (11.8%)	0	51 (11.3%)	36 (53.7%)	
Portal					
None	312 (42.2%)	164 (74.2%)	142 (31.4%)	6 (9.0%)	< 0.001
Ring	32 (4.3%)	16 (7.2%)	16 (3.5%)	0	
Target	241 (32.6%)	26 (11.8%)	195 (43.1%)	20 (29.9%)	
Core	23 (3.1%)	4 (1.8%)	19 (4.2%)	0	
Heterogeneous	132 (17.8%)	11 (5.0%)	80 (17.7%)	41 (61.2%)	
Equilibrium					
None	305 (41.2%)	162 (73.3%)	138 (30.5%)	5 (7.5%)	< 0.001
Ring	32 (4.3%)	15 (6.8%)	17 (3.8%)	0	
Target	240 (32.4%)	28 (12.7%)	191 (42.3%)	21 (31.3%)	
Core	21 (2.8%)	3 (1.4%)	18 (4.0%)	0	
Heterogeneous	142 (19.2%)	13 (5.9%)	88 (19.5%)	41 (61.2%)

### CT features of HEH on per-patient analysis

**Fig. 5:**
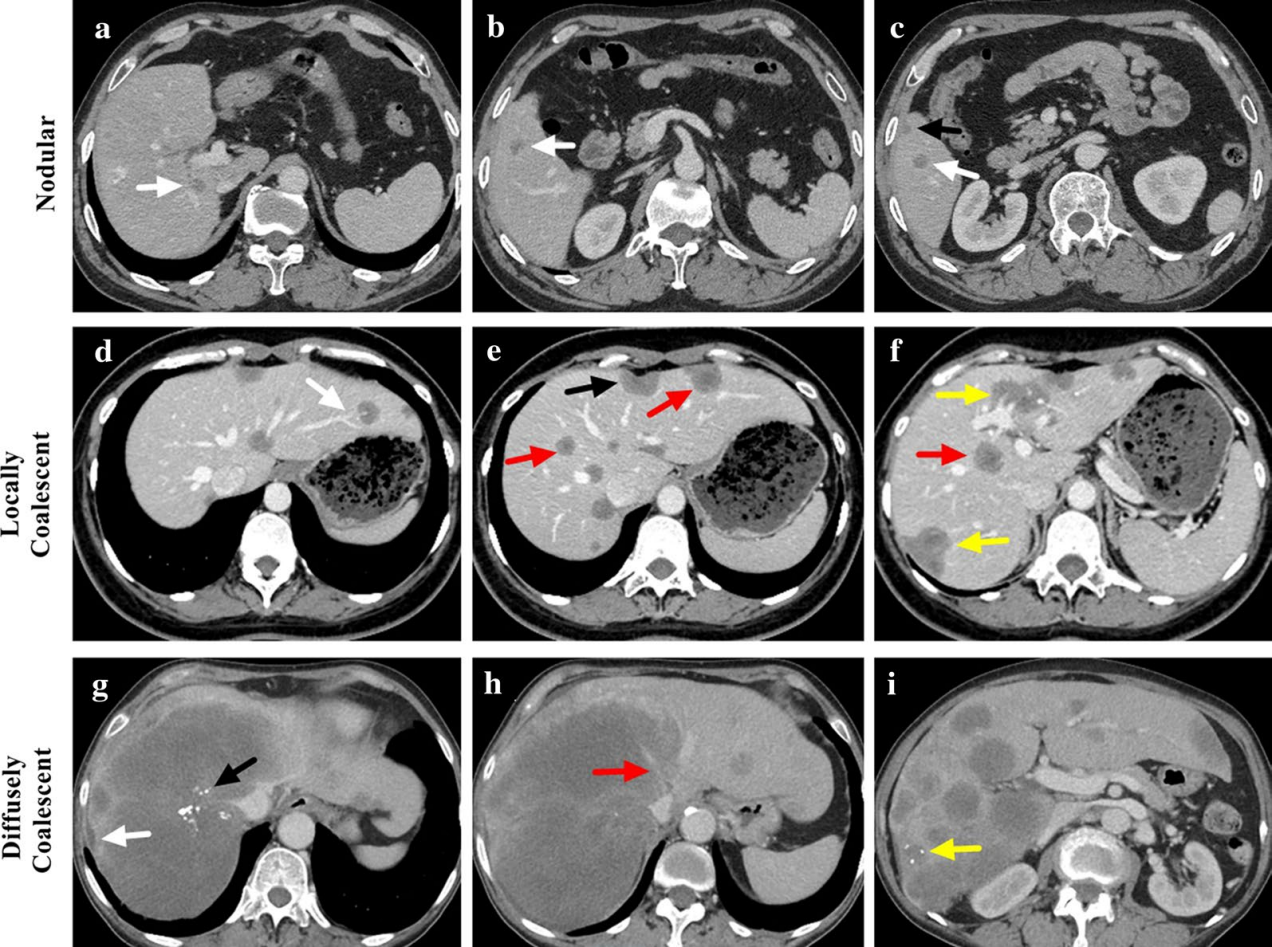
**a–c** Contrast-enhanced CT scan of a 44-year-old male HEH patient in nodular group. Multiple intrahepatic nodular lesions could be found (marked with white arrows) with one subcapsular lesion (marked with a black arrow). No target sign or lollipop sign was detected. **d–f** Contrast-enhanced CT scan of a 32-year-old female HEH patient in locally coalescent group. Locally coalescent lesions could be found (marked with yellow arrows). A subcapsular lesion caused capsular retraction (marked with a black arrow). Both target sign (marked with red arrows) and lollipop sign (marked with a white arrow) could be detected. **g–i** Contrast-enhanced CT scan of a 68-year-old female HEH patient in diffusely coalescent group. Right hepatic vein disappeared, and middle hepatic vein was involved by the diffusely coalescent lesion (marked with a red arrow). Both capsular retraction (marked with a white arrow) and calcification (marked with a black arrow) could be detected. One subcapsular lesion with calcification and capsular retraction was marked with a yellow arrow

According to the type of the largest lesion, patients were categorized into three groups: nodular (*n* = 39, 41.9%), locally coalescent (*n* = 35, 37.6%) and diffusely coalescent (*n* = 19, 20.4%). Patients in locally coalescent group were more likely to have more than 10 lesions, compared with nodular and diffusely coalescent groups (*p* < 0.001) (Table 3). No difference was found in the rate of extrahepatic metastasis among the three groups (*p* = 0.086). The rate of patients with subcapsular lesion was 64.1% in nodular group, while all patients in locally and diffusely coalescent groups had subcapsular lesion. Patients in nodular group had the lowest rate in capsular retraction and lollipop sign; nevertheless, patients in locally coalescent group had the highest rate in lollipop sign and target sign (Fig. 5, Table 3). Capsular retraction, vascular invasion and calcification were mostly detected in patients of diffusely coalescent group (Fig. 5, Table 3). No difference was found in the rate of calcification among the three groups (*p* = 0.732).

**Table 3:** Comparison of CT features among HEH patients with different lesion types

Parameters	Total (*n* = 93)	Nodular (*n* = 39)	Locally Coalescent (*n* = 35)	Diffusely Coalescent (*n* = 19)	*p* value
Number of Lesions					
Singular	3 (3.2%)	3 (7.7%)	0	0	< 0.001
2–10	38 (40.9%)	25 (64.1%)	6 (17.1%)	7 (36.8%)	
More than 10	52 (55.9%)	11 (28.2%)	29 (82.9%)	12 (63.2%)	
Extrahepatic Metastasis					
Yes	48 (51.6%)	18 (46.2%)	23 (65.7%)	7 (36.8%)	0.086
No	45 (48.4%)	21 (53.8%)	12 (34.3%)	12 (63.2%)	
Subcapsular Lesion					
Yes	79 (84.9%)	25 (64.1%)	35 (100%)	19 (100%)	< 0.001
No	14 (15.1%)	14 (35.9%)	0	0	
Capsular Retraction					
Yes	49 (52.7%)	5 (12.8%)	25 (71.4%)	19 (100%)	< 0.001
No	44 (47.3%)	34 (87.2%)	10 (28.6%)	0	
Lollipop Sign					
Yes	49 (52.7%)	15 (38.5%)	26 (74.3%)	8 (42.1%)	0.005
No	44 (47.3%)	24 (61.5%)	9 (25.7%)	11 (57.9%)	
Target Sign					
Yes	71 (76.3%)	26 (66.7%)	33 (94.3%)	12 (63.2%)	0.006
No	22 (23.7%)	13 (33.3%)	2 (5.7%)	7 (36.8%)	
Vascular Invasion					
Yes	41 (44.1%)	1 (2.6%)	21 (60.0%)	19 (100%)	< 0.001
No	52 (55.9%)	38 (97.4%)	14 (40.0%)	0	
Calcification					
Yes	7 (7.5%)	2 (5.1%)	3 (8.6%)	2 (10.5%)	0.732
No	86 (92.5%)	37 (94.9%)	32 (91.4%)	17 (89.5%)

### Comparison of clinical data among HEH patients with different lesion types

**Fig. 6:**
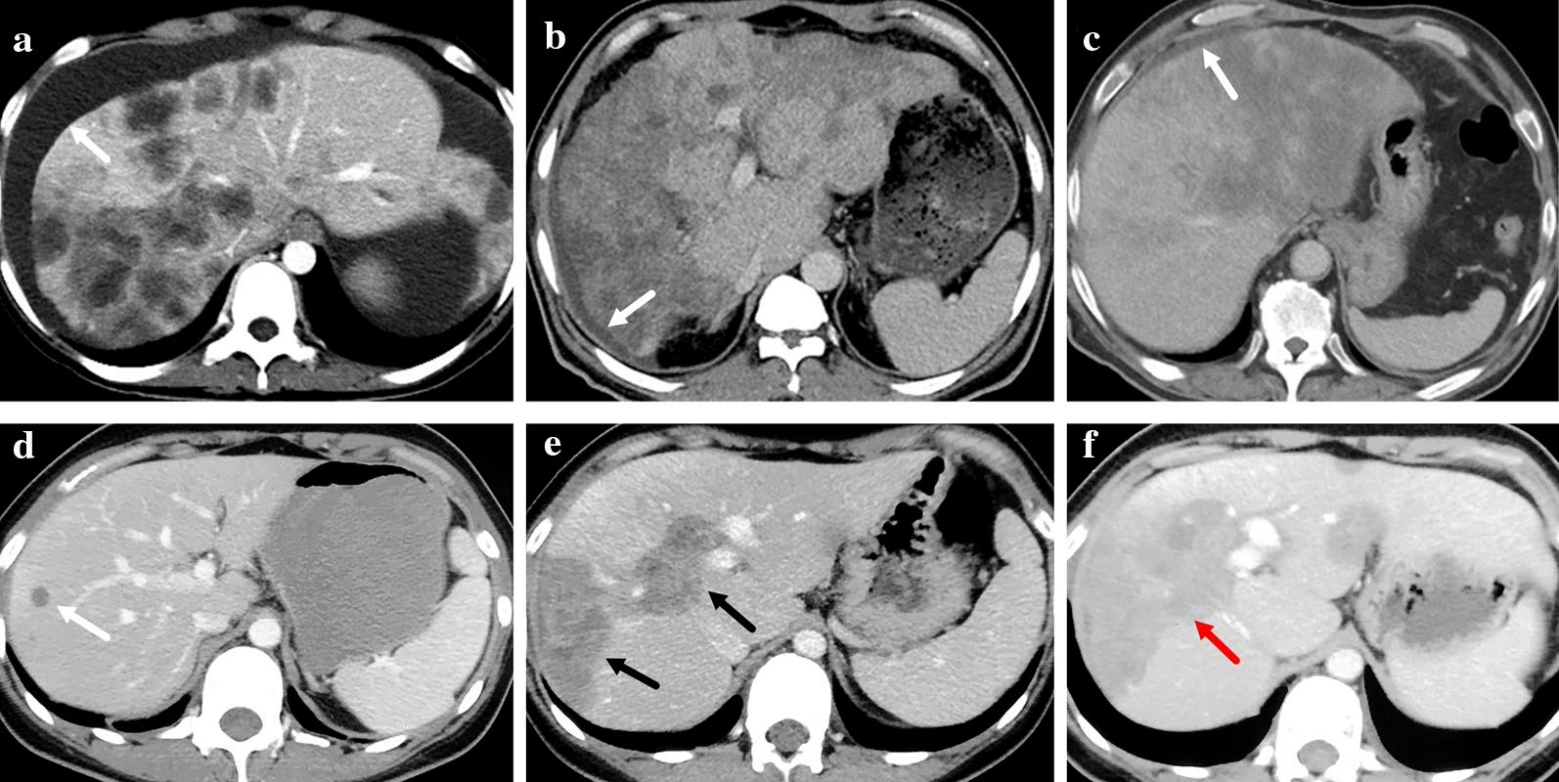
**a–c** Perihepatic effusion of three HEH patients on CT scan (marked with white arrows). **d–f** The changes of CT appearances of a 28-year-old male HEH patient. During the period of 6 years follow-up, the patient received no treatment. **d** At the time of diagnosis, nodular lesion could be found (marked with a white arrow). **e** The CT scan at 4 years after the diagnosis showed locally coalescent lesions (marked with black arrows). **f** The CT scan at 6 years after the diagnosis showed diffusely coalescent lesion (marked with a red arrow)

Compared with patients in nodular and locally coalescent groups, patients in diffusely coalescent group had the highest rate of abdominal pain (42.1%) (*p* = 0.006) and perihepatic effusion (52.6%) (*p* < 0.001) (Fig. 6, Table 4). Patients in diffusely coalescent group also had the highest serum levels of alanine aminotransferase (ALT), aspartate aminotransferase (AST), alkaline phosphatase (ALP) and gamma-glutamyl transpeptidase (GGT) (Table 4). Compared with patients in nodular and locally coalescent groups, patients in diffusely coalescent group had the highest rate of abnormality in liver function (defined as serum abnormality in any parameter, including ALT, AST, ALP and GGT) (*p* < 0.001) (Table 4).

**Table 4:** Comparison of clinical data among HEH patients with different lesion types

Parameters	Total (*n* = 93)	Nodular (*n* = 39)	Locally Coalescent (*n* = 35)	Diffusely Coalescent (*n* = 19)	*p* value
Age	39.4 ± 13.9	39.6 ± 12.2	35.7 ± 11.8	45.7 ± 18.2	0.037
Gender					
Male	42 (45.2%)	21 (53.8%)	16 (45.7%)	5 (26.3%)	0.141
Female	51 (54.8%)	18 (46.2%)	19 (54.3%)	14 (73.7%)	
Abdominal Pain					
Yes	17 (18.3%)	3 (7.7%)	6 (17.1%)	8 (42.1%)	0.006
No	76 (81.7%)	36 (92.7%)	29 (82.9%)	11 (57.9%)	
Perihepatic Effusion					
Yes	10 (10.8%)	0	0	10 (52.6%)	< 0.001
No	83 (89.2%)	39 (100%)	35 (100%)	9 (47.4%)	
ALT (IU/L)	32.4 ± 21.6	27.2 ± 16.2	29.5 ± 14.9	48.5 ± 32.5	0.001
AST (IU/L)	29.6 ± 16.9	24.3 ± 10.7	28.3 ± 12.4	42.7 ± 26.0	< 0.001
ALP (IU/L)	110.1 ± 71.9	83.2 ± 37.2	113.5 ± 67.4	162.0 ± 102.3	< 0.001
GGT (IU/L)	43 ± 70	37 ± 26	41 ± 91	123 ± 150	< 0.001
Abnormality in Liver Function					
Yes	34 (36.6%)	8 (20.5%)	12 (34.3%)	14 (73.7%)	< 0.001
No	59 (63.4%)	31 (79.5%)	23 (65.7%)	5 (26.3%)	

Abnormality in liver function was defined as serum abnormality in any parameter, including ALT, AST, ALP and GGT

## Discussion

HEH is an extremely rare liver tumor and patients could have different long-term prognoses due to the disparity in tumor malignancy [14, 15]. Although the tumor sometimes behaved with severe malignancy, spontaneous tumor regression has been observed in some cases [3, 16, 17]. Surgical resection and liver transplantation have been deemed as an optional treatment choice for HEH with good long-term survival [18–22]. Although IFN-a 2b has been reported to be an effective treatment used as monotherapy or combined with target therapy [5, 9, 23, 24], no standard treatment paradigm has been established.

Several studies have reported the radiological features of HEH, such as subcapsular lesion, coalescent lesion, capsular retraction, lollipop sign and target sign [25, 26]. Lollipop sign was depicted as hepatic or portal vein tapering and terminating at the edge of a well-defined peripherally lesion, which was previously reported to be a characteristic feature of HEH [13, 27]. Target sign which was depicted as two or multiple concentric layered “target-like” appearance was also reported to be a specific radiological feature of HEH [13, 28–30]. However, target sign could also appear on the radiological image of patients with hepatic metastatic tumor (HMT) [8]. The results of our previous study showed that capsular retraction and lollipop sign were more specific MRI features of HEH, which could be used for distinguishment from HMT [8].

In this study, a total of 740 lesions were evaluated and the per-lesion analysis showed that CT features differed significantly among lesions of different sizes, which was similar to the results of our previous study on MRI [8]. Most of the evaluated lesions were nodular and coalescent lesions accounted for about 20% of lesions. We speculate that nodular lesion was the lesion in the early stage and locally coalescent lesion which was formed by the fusion of adjacent lesions as they grew, represented the progression of the disease. Diffusely coalescent lesion occupying multiple liver segments may indicate the advanced stage of the disease. The rates of subcapsular lesion and capsular retraction increased as lesion size increased. The rate of lollipop sign was highest in the medium group, which was inconsistent with our previous reports, and the reason could be the differences on the definition of lollipop sign. Both lollipop sign and vascular invasion depicted the relationship between lesion and vascular, while in our previous study, we didn’t evaluate the feature of vascular invasion. In this study, vascular invasion was defined as that portal or hepatic vein penetrated lesions or was surrounded by lesions, which may be classified as lollipop sign in our previous study. From our point of view, both lollipop sign and vascular invasion represented the closely relationship between tumor and portal or hepatic vein, and the total rate of them was similar to our previous report [8].

The enhancement pattern of HEH also differed greatly among lesions of different sizes. For small lesions (< 2 cm), most of them had no enhancement in arterial, portal and equilibrium phases. Lesions in medium group (2–5 cm) had the highest rate of target-like enhancement, while large lesions (> 5 cm) were more likely to present heterogenous enhancement. Sometimes, target sign could not only be detected on contrast-enhanced CT images but also on unenhanced CT scans, which could be used as a hint of HEH. Consistently, HEH tumors were pathologically described as having a prominent stroma with abundant fibrous connective tissue, and HEH was considered as in the range of fibrous tumor. So, the radiological appearances of HEH lesions such as target sign and capsular retraction could represent the common characteristics of tumors with fibrous phenotype. Calcification in the HEH lesion was also found in previous studies [25, 31, 32]. In this study, only 15 lesions (2.0%) showed calcification and the rate was highest in the large group.

Based on the type of largest lesion, HEH patients were categorized into three groups in this study. On per-patient analysis, the rates of lollipop sign and target sign were 52.7% and 76.3%, respectively, and patients in locally coalescent group had the highest rates of them (74.3% and 94.3%, respectively). All patients in diffusely coalescent group presented capsular retraction and vascular invasion. For patients in nodular group, the rates of capsular retraction, lollipop sign and target sign were 12.8%, 38.5% and 66.7%. Thus, sometimes HEH patients with nodular type may fail to present any CT characteristics, which could lead to the misdiagnosis. As the growth of nodular lesions, locally coalescent lesion could be formed by the fusion of adjacent lesions. At this stage, patients were more likely to present the characteristics of HEH, such as target sign and lollipop sign. Instead of developing from one lesion, diffusely coalescent lesion originated from widely coalescence of multiple lesions, which could be observed on the CT changes of HEH patient with long-term observation (Fig. 6). Comparison of clinical data showed that patients in diffusely coalescent group had the highest rates of abdominal pain and abnormalities in liver function. Moreover, perihepatic effusion could only be found in patients in diffusely coalescent group. According to previous studies, clinical symptom and effusion were both related to the poor prognosis of EH [14, 33]. So, the existence of diffusely coalescent lesion may have the value of indicating the advanced stage of the disease. Considering the discrepant radiological characteristics among different types, CT appearances of HEH should be classified into nodular type, locally coalescent type and diffusely coalescent type.

There are two major limitations about this study. Firstly, CT examinations of included patients were not conducted in the same institution. Although strict scrutiny on the quality of CT images was conducted, the differences in contrast material and equipment could still affect the CT appearances. However, due to the rarity of the disease, the current study still provided the most comprehensive review of CT appearances of HEH. Secondly, we speculated that lesion types of HEH may correlate with the severity of the disease, but the prognosis was not analyzed. The main reasons were the short term of follow-up for many patients and the diversified treatment modalities, which could obviously impact the prognosis.

In conclusion, CT appearances of HEH differed significantly among lesions of different sizes and types. Patients in locally coalescent group had the highest rate of target sign and lollipop sign, while all patients in diffusely coalescent group had subcapsular retraction and vascular invasion. Considering the differences in characteristics, CT appearances of HEH should be classified into nodular type, locally coalescent type and diffusely coalescent type.

## Data Availability

The data used during this study are available from the corresponding author on reasonable request.

## Abbreviations

ALP: Alkaline phosphatase
ALT: Alanine aminotransferase
AST: Aspartate aminotransferase
CT: Computed tomography
EH: Epithelioid hemangioendothelioma
GGT: Gamma-glutamyl transpeptidase
HEH: Hepatic epithelioid hemangioendothelioma
HMT: Hepatic metastatic tumor
IFN-a 2b: Interferon-alpha 2b
MRI: Magnetic resonance imaging
PACS: Picture archiving and communication system
